# Modulation of Wnt/β-catenin signaling in IL-17A-mediated macrophage polarization of RAW264.7 cells

**DOI:** 10.1590/1414-431X20209488

**Published:** 2020-06-19

**Authors:** Chao Yuan, Dandan Yang, Jia Ma, Jiali Yang, Jing Xue, Fuyang Song, Xiaoming Liu

**Affiliations:** 1 Key Laboratory of Ministry of Education for Conservation and Utilization of Special Biological Resources in the Western China, Yinchuan, China; 2 College of Life Science, Ningxia University, Yinchuan, Ningxia, China; 3 Department of Anatomy and Cell Biology, University of Iowa, Iowa City, IA, USA

**Keywords:** Wnt/β-catenin signaling, Interleukin-17A, Macrophage polarization, STAT, Immune regulation

## Abstract

Macrophages play pivotal roles in host defense and immune homeostasis, which have two major functional polarization states, the classically activated M1 and the alternatively activated M2. Interleukin (IL)-17A is an immune modulator able to shape macrophage phenotypes. Wnt/β-catenin is a developmental signaling pathway that plays crucial roles in morphogenesis and tissue homeostasis, which has also been recently demonstrated playing roles in immune regulation. A growing amount of evidence suggests that both Wnt and IL-17A signaling are involved in macrophage polarization. However, their interaction in macrophage polarization remains elusive. The aim of present study was to explore impacts of Wnt/β-catenin on IL-17A-mediated macrophage M1/M2 polarization in murine monocyte/macrophage-like cell line RAW264.7. Results revealed that IL-17A activated Wnt/β-catenin signaling and induced macrophage M1 polarization, but inhibited M2 polarization. In contrast, the activation of Wnt/β-catenin signaling led to the inhibition of M1 macrophage polarization but the promotion of M2 polarization. Importantly, the activation of Wnt/β-catenin also showed abilities to inhibit the IL-17A-induced M1 macrophage polarization while diminishing the IL-17A-inhibited M2 polarization. Molecular analysis further uncovered that the JAK/STAT signaling pathway was involved in the interaction of Wnt/β-catenin and IL-17A in the modulation of macrophage polarization. These results suggested that the Wnt/β-catenin signaling modulated IL-17A-altered macrophage polarization in part by regulating the JAK/STAT signaling pathway. This study thus revealed a novel function of Wnt/β-catenin signaling in regulating IL-17A-altered macrophage polarization.

## Introduction

Macrophages are immune cells that play key roles in inflammatory and maintenance of immune homeostasis. In this regard, macrophages are able to eradicate a variety of pathogenic microorganisms by phagocytosis in the body (1), where they are implicated in the regulation of inflammatory occurrence and development by producing pro-inflammatory/anti-inflammatory factors in response to an infection and/or insult stress (2).

Taxonomically, owing to the plasticity of macrophages to integrate various dangerous signals, macrophages can be classified as M1 and M2 macrophages based upon their polarizations, i.e., how macrophages are spatially and temporally activated to adopt different functional programs in response to diverse signals, such as microbial and insults or stresses of resident tissues (3). Functionally, M1 macrophages are characterized as a classic activated macrophage phenotype. They are typically polarized by Th1 cytokines such as interferon-γ or lipopolysaccharide, and initiate an immune response by producing pro-inflammatory cytokines, reactive oxygen intermediates, and nitric oxide, phagocytizing microbes. Therefore, M1 macrophages generally play a pro-inflammatory role in response to microbial infections by producing interleukin (IL)-1β, IL-6, tumor necrosis factor (TNF)-α, and other chemokines (4). However, an excessive activation of M1 macrophages may also cause damage and disturbances of homeostasis in their resident tissues (5).

On the other hand, M2 macrophages are induced by certain Th2 cytokines such as IL-4, IL-10, or IL-13, which have a phenotype that more likely adapts to an anti-inflammatory role and tissue repair (6). Phenotypically, M1 macrophages can be characterized by the expression of markers CD86, inducible nitric oxide synthase (iNOS) (7). The M2 polarization of macrophages can be identified by the expression of surface CD206 and effectors contributing to resolution of inflammation and tissue modeling such as IL-10, transforming growth factor (TGF)-β, Arg1, and Ym1 (8).

A compelling body of evidence has shown that macrophage polarization can be induced by a variety of factors, including IL-17A (9). IL-17A, also known as IL-17, is the prototype pro-inflammatory cytokine of the IL-17 family, which is largely produced by the Th17 T helper cell subset, a recently identified subset of effector Th cells (10). In addition to Th17 cells, γδT cells and macrophages are also capable of producing IL-17A (11). Functionally, IL-17A acts as a pro-inflammatory cytokine with differential effects on innate immune cells. It can recruit monocytes in the injury/infected sites where monocytes are differentiated into macrophages and substantially produce cytokine/chemokine to mediate a link between acquired and innate immunity by altering the polarization phenotype of macrophages (9,12), although it is not required for classical macrophage activation in some circumstances (13). Functions of IL-17A have been extensively investigated in chronic inflammatory diseases, such as autoimmune disease and atherosclerosis (14). With respect to macrophage polarization, IL-17A has been reported to enhance both M1 and macrophage-polarizing signals *in vivo* and *in vitro* (15).

Interestingly, studies recently demonstrated that Wnt/β-catenin, a crucial developmental signaling, plays a key role in the development of immune system, but also immune regulation in response to stimuli, particularly its associations with inflammation in macrophages and epithelial cells, which have created a considerable interest in immune-related Wnt functions (16). Several studies have uncovered that the Wnt/β-catenin signaling could effectively exacerbate IL-4- or TGF-β1-mediated macrophage M2 polarization, and blocked β-catenin signaling inhibited the macrophage M2 polarization (17). Wnt/β-catenin signaling promoted macrophage polarization and proliferation was associated with kidney fibrosis (17). Of great interest, Wnt/β-catenin signaling showed an ability to mediate the alteration of macrophage polarization states induced by various factors (18). For instance, the activating transcription factor 3 (ATF3) could promote macrophage M2 polarization state but inhibit the M1 phenotypic alteration by activation of tenascin (TNC), along with the activation of Wnt/β-catenin signaling. Importantly, the ATF3-induced TNC was tightly regulated by Wnt/β-catenin signaling, suggesting that ATF3-altered macrophage polarization was mediated by Wnt/β-catenin signaling pathway (18).

In view of the above findings, we hypothesized that the Wnt/β-catenin signaling might also be involved in the regulation of IL-17A-altered macrophage polarization. The objective of this study was thus to investigate the function of Wnt3A, a ligand of canonical Wnt/β-catenin signaling in IL-17A-induced macrophage polarization in murine macrophage-like RAW264.7 cells.

## Material and Methods

### Cell lines and Wnt3a conditioned medium

The mouse monocyte/macrophage-like cell line RAW264.7 (ATCC#TIB-71), the Wnt3a producing cell line, L cells expressing Wnt3a (overexpression mouse Wnt3a ATCC#CRL-2647), and its parent L cell line (ATCC#CRL-2648) were obtained from American Type Culture Collection (USA). The Wnt3a-expressing and its parent L cell lines were cultured in DMEM medium (Gibco, USA) containing 10% fetal bovine serum (FBS) and 1% penicillin/streptomycin at 37°C in 5% CO_2_. When both Wnt3a and the parent L cell lines were grown to 70−80% confluence, cells were refreshed with DMEM/5% FBS, then the culture media were collected and refreshed at 24, 48, and 72 h. The collected media were pooled and centrifuged at 5000 *g* for 10 min at 4°C and filtered with 0.22-µm pore filters prior to being aliquoted and stored at -80°C until use. The conditioned media collected from Wnt3a-expressing and parent control L cells were designated as Wnt3a-CM and Ctrl-CM in this study, respectively.

### Reagents and antibodies

Recombinant human IL-17A was purchased from PeproTech (USA). Rat PE-conjugated anti-mouse CD86 and rat FITC-conjugated anti-mouse CD206 were purchased from BioLegend (USA). Rabbit anti-GSK-3β, Arg1, β-catenin, active-β-catenin (ABC), phospho-STAT1 (signal transducers and activators of transcription 1), and phospho-STAT3 antibodies were products of Cell Signaling Technology (USA). Rabbit anti-STAT6 and phosphor-STAT6 antibodies were purchased from Affinity Biosciences (USA). Rabbit anti-iNOS antibody was purchased from Abcam (USA), rabbit anti-p21 was a product of Santa Cruz Biotech (USA). Rabbit anti-STAT3, SOCS3, BCL-XL, c-Myc, TCF-4, β-actin, and mouse anti-Cyclin D1 antibodies were purchased from Proteintech (China). The Wnt signaling inhibitor XAV939 was purchased from Santa Cruz Biotech.

### Cell culture and treatment

RAW264.7 cells were cultured in DMEM containing 10% FBS at 37°C in 5% CO_2_. The cells were resuspended in DMEM containing 10% FBS and seeded to a six-well plate until the cells adhered to the plate after 6 h. The medium of RAW264.7 cells was replaced with 1 mL of fresh DMEM containing 10% FBS and 1 mL conditional medium (CM) with/without 50 ng/mL of rIL-17A and 2 μM XAV939. After 24 h, the supernatant of RAW264.7 cells was collected for ELISA. The cells were harvested for protein isolation and subjected to Western blotting assay.

### Western blotting analysis

Total cell-protein samples were analyzed. The cells were lysed with Whole Cell Lysis buffer (KeyGEN, China) and kept for 60 min on ice. Then, the lysates were centrifuged at 12,000 *g* for 15 min at 4°C and the supernatants were collected as whole cell extracts. The concentration of protein was determined using a BCA Assay kit (KeyGEN, China) and were solubilized in 6× protein buffer (TransGen Biotech, China). The protein (60 μg) was loaded and resolved in a 10% sodium dodecyl sulfate-polyacrylamide gel (SDS-PAGE), before it was transferred to polyvinylidene difluoride (PVDF) membranes (Millipore, USA). Membranes were blocked in 5% skimmed milk in PBS containing 0.2% Tween-20 and incubated with appropriate primary antibodies to proteins of interest overnight at 4°C. After washing with PBS-0.1% Tween-20 (PBST), membranes were incubated with horseradish peroxidase-conjugated goat anti-rabbit secondary antibodies (ThermoFisher, USA) for 2 h at room temperature. The membranes were then developed with an ECL detection system (PerkinElmer, USA) for proteins of interest. The abundance of protein expression was semi-quantified by optical densitometry using ImageJ Software version 1.46 (https://rsb.info.nih.gov/ij/). The ratio of the net intensity of each sample was normalized by the β-actin internal control and was calculated as densitometric arbitrary units (A.U.), which served as an index of relative expression of a protein of interest.

### Real-time PCR

Total RNA from cultured RAW264.7 cells was isolated using Trizol reagent (Invitrogen, USA) and subsequently used for cDNA synthesis according to manufacturer’s instructions (TaKaRa, Japan). The quantitative real-time RT-PCR was performed in the QuantStudio 5 system (Thermo Fisher Scientific, USA) using a SYBR Green 1 kit (TaKaRa). The primer sets used in this study are listed in Table 1.

**Table 1:** Primer sequences for real-time PCR analysis.

Gene	GenBank number	Sequence (5′-3′)	Tm (°C)	Size (bp)
GAPDH	NM_008084.3	F: CCATGTTTGTGATGGGTGTGAACCA	54.0	251
		R: ACCAGTGGATGCAGGGATGATGTTC		
MCP-1	NM_011333.3	F: CTCGGACTGTGATGCCTTAAT	54.15	106
		R: TGGATCCACACCTTGCATTTA		
Fizz1	NM_020509.3	F: CGTGGAGAATAAGGTCAAGGA	55.54	134
		R: CAGTAGCAGTCATCCCAGCA		
Ym-1	NM_009892.3	F: TCTCTACTCCTCAGAACCGTCAG	55.9	160
		R: CGCATTTCCTTCACCAGAAC		

F: forward; R: reverse; Tm: temperature.

### ELISA analysis

The concentration of inflammatory factors IL-6, IL-10, and TNF-α in the supernatants was measured by ELISA using commercially available kits according to manufacturer’s instructions (Boster Biological Technology, China). Briefly, samples were added to each well and incubated at 37°C for 90 min. Then, 100 μL biotin-labeled anti-mouse IL-6, IL-10, and TNF-α were added and incubated at 37°C for additional 1 h. The wells were washed with washing buffer 3 times. Then, affinity peroxidase complex was used as the secondary antibody. After 30-min incubation, the wells were extensively washed 5 times, followed by the addition of 90 μL TMB and incubation for 15 min before 100 μL of stopping solution was added to each well. The absorbance was measured at 450 nm (OD_450nm_) and then converted into a concentration (ng/mL) through a standard curve.

### Flow cytometry assay

RWA264.7 cells (2×10^6^) treated with varied conditions were dissociated with trypsin and collected, washed twice in PBS prior to being suspended in PBS at 2×10^6^ cells/mL. Non-specific binding was blocked by incubating the cells with 1.0 µg of TruStain FcX antibody (Biolegend, Cat. #101319) per 2×10^6^ cells in 200 µL volume for 30 min on ice. The cells were then washed in PBS for 3×5 min before they were incubated with fluorescently labeled antibodies, CD86-PE (Biolegend, Cat. #105008), and CD206-FITC (Biolegend, Cat. #141704) for an hour at room temperature. Then, cells were washed twice in PBS prior to performing flow cytometry assay in a flow cytometry system (Becton and Dickinson, USA) and analyzed using FlowJo software (TreeStar, Inc., USA).

### Data analysis

The data are reported as means±SD for at least three independent experiments for each condition. All data were analyzed using PRISM 5 (GraphPad Software, USA). Statistical evaluation of the data was performed by one-way ANOVA when more than two groups were compared with a single control or comparison of differences between two groups. P values <0.05 were considered to be significant.

## Results

### IL-17A activated canonical Wnt/&mac_bgr;-catenin signaling in RAW264.7 cells

**Figure 1:**
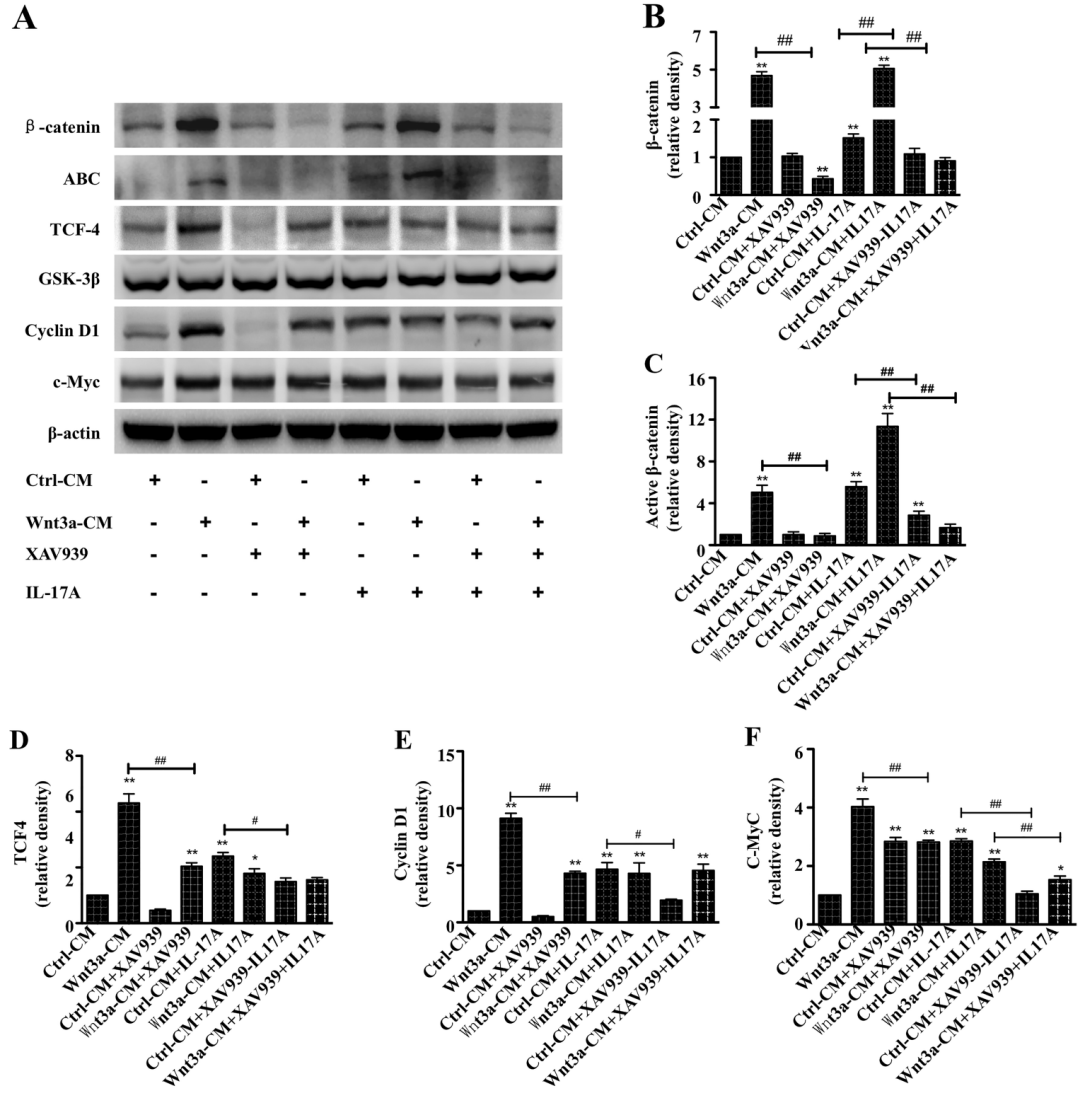
Interleukin (IL)-17A activated Wnt/β-catenin signaling in murine macrophage RAW264.7 cells. **A**, Representative images of immunoblots of indicated proteins of interest in cells treated with different conditions. Semi-quantitative analysis of the expression of proteins in (**A**) showing fold-changes of protein abundance of β-catenin (**B**), active β-catenin (**C**), TCF-4 (**D**), Cyclin D1 (**E**), and c-MyC (**F**) in cells treated with Wnt3a-CM and/or XAV939 in the presence or absence of rhIL-17A as determined by densitometry assay using ImageJ software Fiji. Wnt3a-CM and rhIL-17A synergistically activated Wnt/β-catenin signaling in RAW264.7 cells. Data are reported as means±SD from three independent experiments. *P<0.05, **P<0.01 compared to the Ctrl-CM group; ^#^P<0.05, ^##^P<0.01 compared to the indicated groups (ANOVA).

As expected, the results depicted in Figure 1 showed an enhanced activation of Wnt/β-catenin signaling, as determined by an increased protein expression of global β-catenin (Figure 1A and B), the active form of β-catenin (ABC) (Figure 1A and C), transcriptional factor TCF-4 (Figure 1A and D), and Wnt/β-catenin signaling targeted genes, Cyclin D1 (Figure 1A and E) and c-Myc (Figure 1A and F), in cells exposed to Wnt3a-CM compared to Ctrl-CM (P<0.01). In contrast, the presence of XAV939 inhibited the Wnt/β-catenin signaling activity along with a decreased expression of the above examined proteins in RAW264.7 cells. Of note, the XAV939-inhibited Wnt activity could be partially restored by Wnt3a-CM (P<0.01). Interestingly, an enhanced activation of Wnt/β-catenin signaling was observed in RAW264.7 cells treated with rhIL-17A, as determined by the expression of active β-catenin and target genes of Wnt/β-catenin signaling, and the rhIL-17A-triggered Wnt signaling activity could be diminished by XAV939 (P<0.01). The addition of rhIL-17A synergistically increased the abundance of active β-catenin in the Wnt3a-CM-treated cells (P<0.01); however, the addition of IL-17A decreased the abundance of several proteins of target genes of Wnt/β-catenin, including TCF-4, Cyclin D1, and c-Myc, compared to cells cultured in Wnt3a-CM alone (Figure 1D–F). These data suggested that IL-17A could modulate the canonical Wnt signaling activity in RAW264.7 cells.

### Wnt3a inhibited IL-17A-induced M1 polarization and reduced IL-17A-repressed M2 polarization

**Figure 2:**
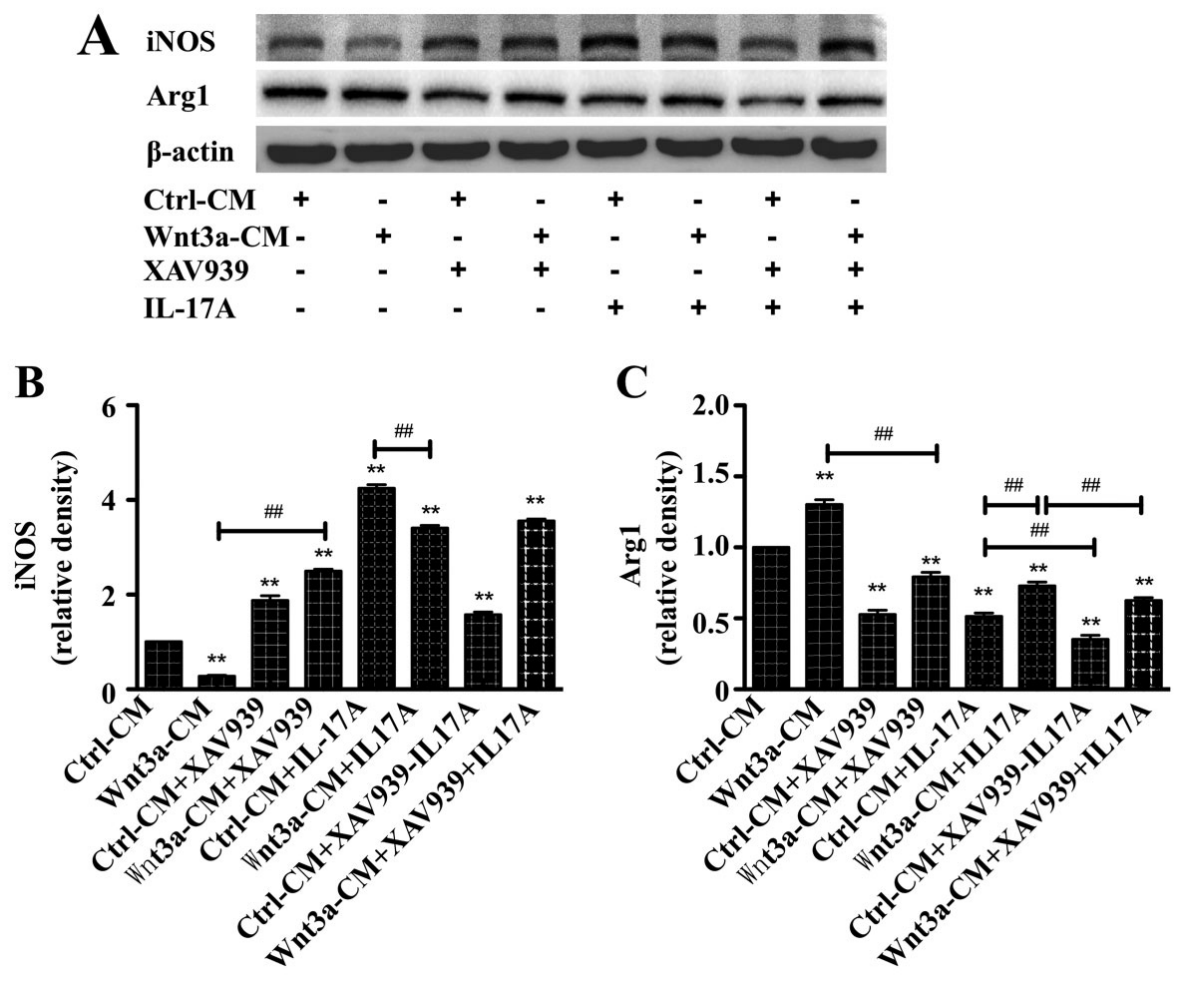
Interleukin (IL)-17A reversed the Wnt3A-altered expression of macrophage polarization markers in murine macrophage RAW264.7 cells. **A**, Representative images of immunoblots of Arg1 and iNOS in cells treated with different conditions. **B** and **C**, Semi-quantitative analysis of the expression of proteins in (**A**) showing fold-changes of Arg1 and iNOS in cells treated with Wnt3a-CM and/or XAV939 with or without rhIL-17A as determined by densitometry assay using ImageJ software Fiji. Data are reported as means±SD from three independent experiments. **P<0.01 compared to the Ctrl-CM group; ^##^P<0.01 compared to the indicated groups (ANOVA).

**Figure 3:**
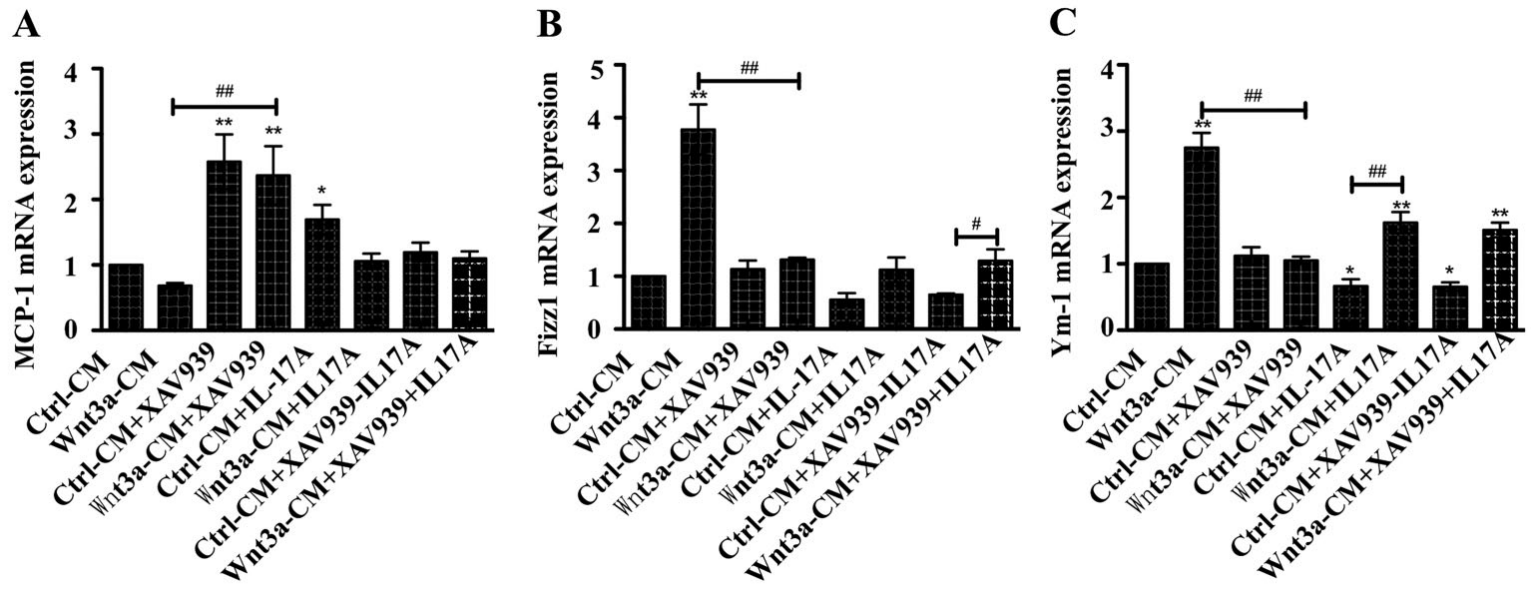
Expression of key macrophage characteristic genes in RAW264.7 cells in response to Wnt3A and/or IL-17A stimulation. **A-C**, Relative expression of *Mcp1*, *Fizz1*, and *Ym-1* genes of the fold-change over control of transcript in cells treated with Wnt3a-CM and/or XAV939 without rhIL-17A or with rhIL-17A. Data are reported as means±SD from three independent experiments. *P<0.05, **P<0.01 compared to the Ctrl-CM group; ^#^P<0.05, ^##^P<0.01 compared to the indicated groups (ANOVA).

The immunoblotting assay showed increased and decreased abundance, respectively, of M2 macrophage marker Arg1 and M1 macrophage marker iNOS in cells treated with Wnt3a-CM. The XAV939 exerted opposite effects of Wnt3a-CM, i.e., inhibited Arg1 and induced iNOS expression, which was also able to inhibit the function of Wnt3a in the expression of these proteins. This result indicated that activation of Wnt signaling inhibited macrophage M1 polarization but promoted M2 polarization in RAW264.7 cells. Unlikely seen in Wnt3a-CM-treated cells, Arg1 was decreased and iNOS was increased in RAW264.7 cells exposed to rhIL-17A. Of interest, IL-17A-altered expression of Arg1 and iNOS could be reversed by the presence of Wnt3a (Figure 2). However, XAV939 displayed a capacity to reduce the IL-17A-altered expression of Arg1 and iNOS proteins. The immunoblotting result implied a potential of Wnt3a in modifying IL-17A-altered macrophage polarization. RT-PCR analysis further revealed a reduced transcript of *Mcp-1* gene, a well-known marker for M1 macrophages, and increased transcripts of *Fizz1* and *Ym-1* genes, two markers for M2 macrophages in rhIL-17A-treated RAW264.7 cells (Figure 3). In contrast, Wnt3a-CM exhibited an opposite effect of IL-17A, which induced the expression of *Mcp-1* transcript and suppressed *Fizz1* and *Ym-1* gene expression. This result was consistent with the above findings of immunoblotting analysis.

**Figure 4:**
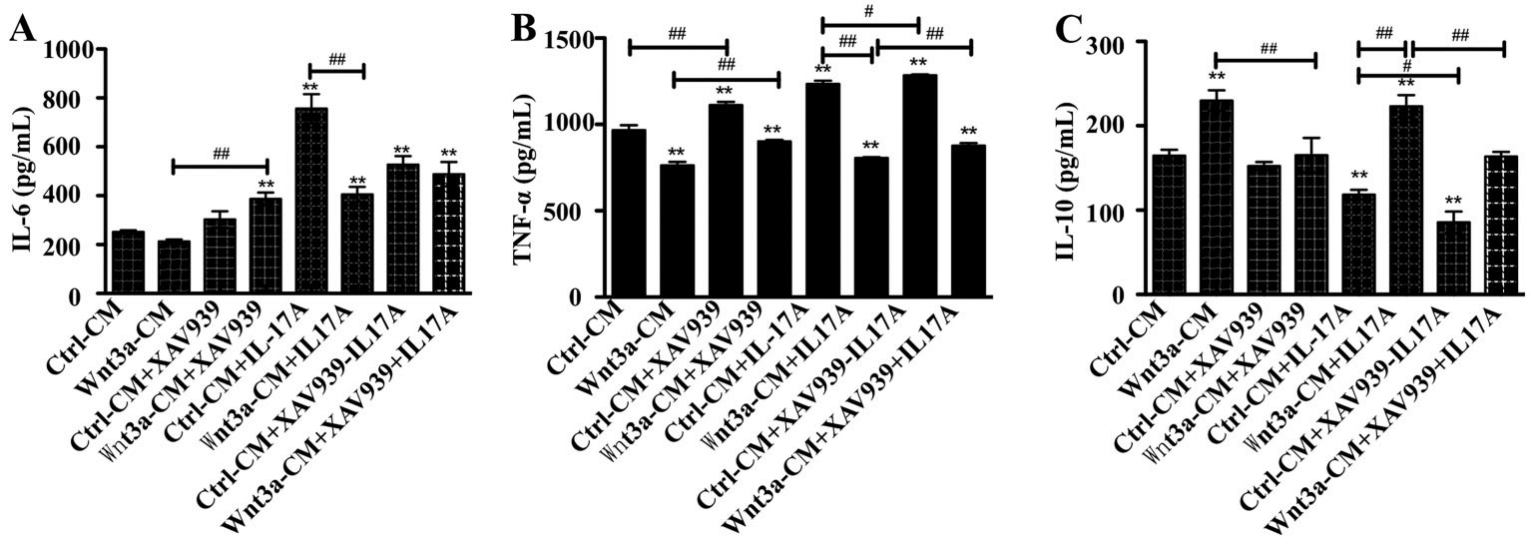
Production of cytokines in RAW264.7 cells in response to Wnt3A and/or interleukin (IL)-17 stimulation. **A–C**, Concentration of IL-6, tumor necrosis factor (TNF)-α, and IL-10 in culture media of cells treated with Wnt3A-CM and/or XAV939 without rhIL-17A or with rhIL-17A. **P<0.01 compared to Ctrl-CM group, ^#^P<0.05, ^##^P<0.01 compared to the indicated groups (ANOVA).

**Figure 5:**
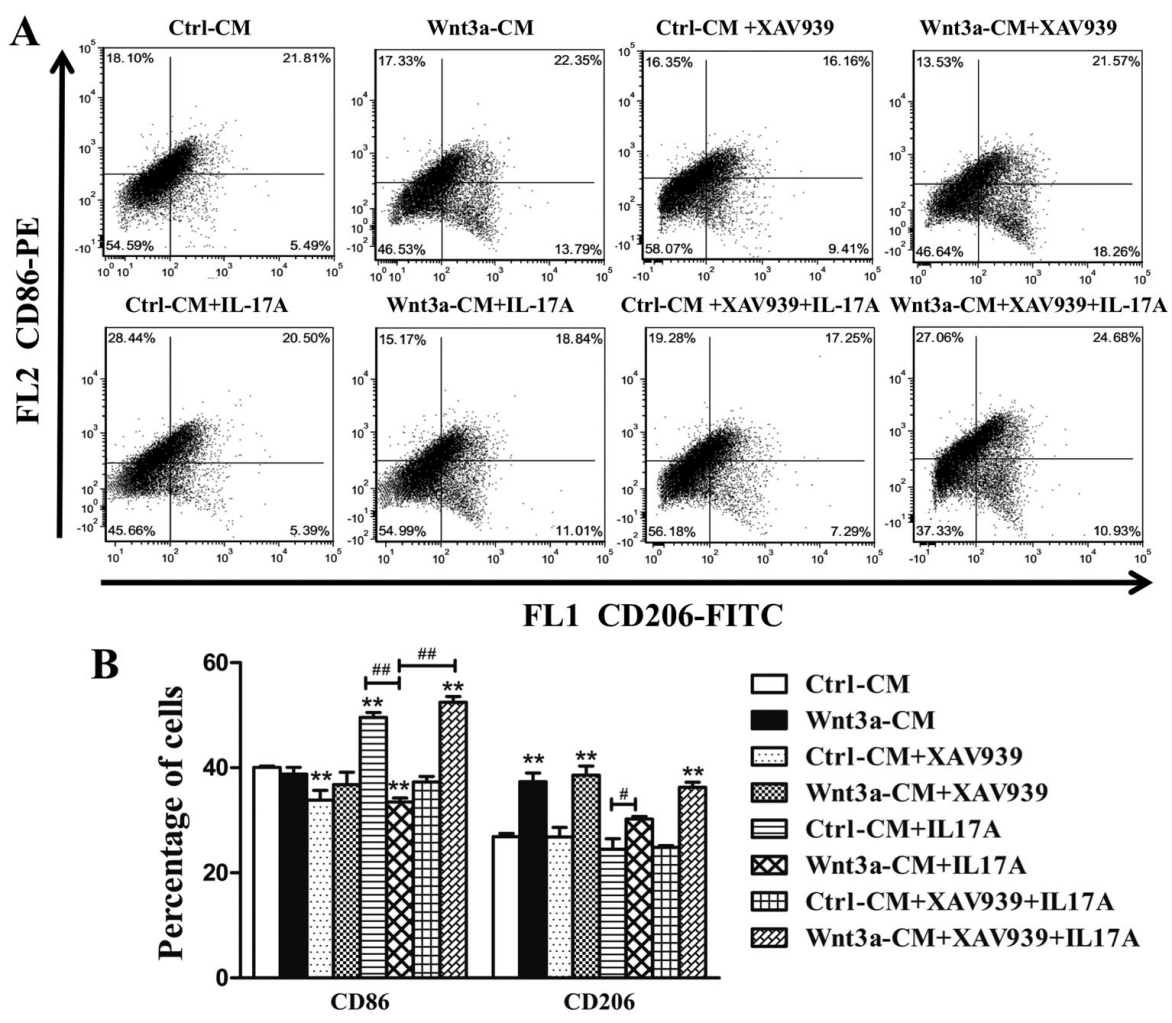
Wnt3a-altered and interleukin (IL)-17A-altered macrophage polarization in murine macrophage RAW264.7 cells determined by the frequency of CD86 and CD206 cell populations. **A**, Representative quadrants of flow cytometry density plots showing distribution patterns of CD86- and CD206-positive cells in samples treated with Wnt3a-CM and/or XAV939 with or without rhIL-17A. **B**, Quantitative analysis of flow cytometry data in **A**. Data are reported as means±SD from three independent experiments. **P<0.01 compared to the Ctrl-CM group; ^#^P<0.05, ^##^P<0.01 compared to the indicated groups (ANOVA).

In agreement with molecular data, the presence of Wnt3a alone reduced the production of Th1 cytokines TNF-α and IL-6, but increased Th2 cytokine IL-10 production in RAW264.7 cells, and the Wnt inhibitor XAV939 could partially offset the effects of Wnt3a. In contrast, the addition of rhIL-17A alone increased the production of TNF-α and IL-6, but reduced IL-10 secretion (Figure 4). The effects of Wnt3a could be partially reversed by IL-17A, and *vice versa*, the functions of IL-17A could be inhibited by Wnt3a. These findings were further phenotypically validated by flow cytometric analysis of M1 macrophage surface marker CD86 and M2 macrophage marker CD206. There were about ∼39.91% of M1 macrophage and ∼27.3% of M2 macrophage subpopulations determined in RAW264.7 cells exposed to Ctrl-CM. The exposure of Wnt3a-CM increased the CD206-positive M2 macrophage population, and rhIL-17A increased the frequency of CD86-positive M1 macrophages in RAW264.7 cells (Figure 5). Interestingly, the rhIL-17A-altered frequencies of M1 and M2 macrophages could be partially reversed by Wnt3a, and the countered effects of Wnt3a were reduced by the addition of Wnt inhibitor XAV939 to some extent. These results clearly suggested that Wnt/β-catenin signaling had an opposite effect of IL-17A and countered the IL-17A-mediated macrophage polarization in RAW264.7 cells.

### STAT signaling was involved in the Wnt3a-modulated IL-17A-altered macrophage polarization

**Figure 6:**
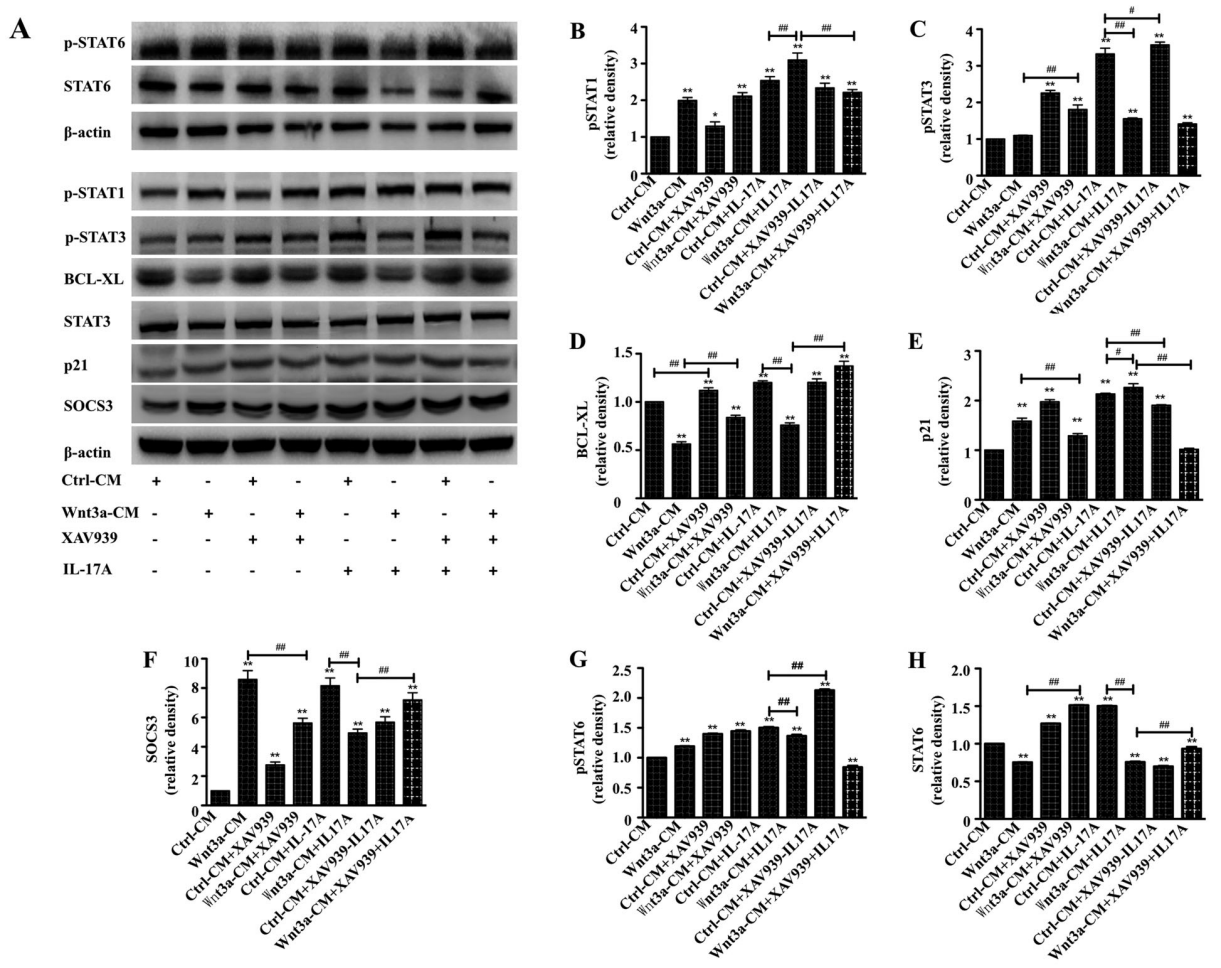
Stat signaling is involved in Wnt3A and IL-17A-mediated macrophage polarization in RAW264.7 cells. **A**, Representative images of immunoblots of indicated proteins of Stat signaling cascade in cells treated with different conditions. Semi-quantitative analysis of the expression of proteins in (**A**) showing fold changes of protein abundance of p-STAT1 (**B**), p-STAT3 (**C**), BCL-XL (**D**), p21 (**E**), SOCS3 (**F**), p-STAT6 (**G**), and STAT6 (**H**) in cells treated with Wnt3a-CM and/or XAV939 in the presence or absence of rhIL-17A determined by densitometry assay using ImageJ software Fiji. Data are reported as means±SD from three independent experiments. *P<0.05, **P<0.01 compared to the Ctrl-CM group, ^#^P<0.05, ^##^P<0.01 compared to the indicated groups (ANOVA).

Macrophage polarization was tightly controlled by the activation of various transcriptional factors, and the regulation of Stat signaling in this procession has been well documented. Janus kinase/signal transducers and activators of transcription (JAK/STAT) signaling activation was thus examined for understanding whether it was involved in the Wnt3a-modulated IL-17A-altered macrophage polarization. As expected, both Wnt3a and IL-17A indeed could enhance Stat signaling by inducing p-STAT1 expression, and Wnt3a could further enhance the expression of IL-17A-induced p-STAT1 in macrophage RAW264.7 cells (Figure 6A and B). Interestingly, Wnt3a and IL-17A showed an opposite effect on the expression of p-STAT3 protein, i.e., Wnt3a inhibited p-STAT3 expression, but IL-17A induced its expression in this cell type (Figure 6A and C). Interestingly, Wnt3a-CM induced STAT6 and IL-17A inhibited the expression (Figure 6H), but both of them increased the phosphorylation of STAT6 protein (Figure 6G). Furthermore, Wnt3a-CM showed a potential to diminish the IL-17A-induced STAT6 protein expression and phosphorylation in RAW264.7 cells (Figure 6). Consistently, a decreased expression of BCL-XL, a downstream gene of Stat signaling cascade, was observed in cells exposed to Wnt3a-CM, but the expression of BCL-XL was increased by rhIL-17A (Figure 6A and D). Of note, both Wnt3a and IL-17A showed an ability to increase the expression of p21, an important member of the cyclin-dependent kinase inhibitors family. The Wnt inhibitor XAV939 could inhibit both Wnt3a- and IL-17A-induced p21 expression. However, XAV939 alone also induced p21 expression in RAW264.7 cells cultured in this condition (Figure 6E), which showed a similar trend to p-STAT1 (Figure 6B). In addition, a highly increased and reduced expression of SOCS3 protein, a suppressor of cytokine signaling, was detected in cells treated with IL-17A and Wnt3a-CM, respectively (Figure 6A and F). Equally noteworthy, less abundant SOCS3 protein was observed in cells treated with a combination of Wnt3a-CM and IL-17A, compared with Wnt3a-CM or IL-17A alone (Figure 6A and F). Moreover, XAV939 alone could also increase the SOCS3 expression but inhibit the Wnt3a- and IL-17A-induced SOCS3 expression (Figure 6A and F), suggesting that Wnt inhibitor XAV939 alone could enhance Stat signaling, which could partially reverse Wnt3a-inhibited Stat signaling activity and synergize IL-17A activity, in a similar trend of effect with IL-17A (Figure 6). These results implied that activation of Wnt/β-catenin pathway inhibited IL-17A-induced macrophage M1 polarization, in part by reducing p-STAT3 expression, while it diminished the IL-17A-suppressed macrophage M2 polarization by augmenting p-STAT1 expression.

## Discussion

In the present study, a regulatory role of Wnt/β-catenin signaling in IL-17A-altered macrophage polarization was explored. The results showed that Wnt/β-catenin signaling could be activated by IL-17A. IL-17A functionally could induce M1 macrophage polarization but repress M2 polarization. Conversely, Wnt3a, a ligand of Wnt/β-catenin, inhibited M1 macrophage polarization but promoted M2 polarization. Importantly, Wnt3a-activated Wnt/β-catenin signaling showed a potential to inhibit IL-17A-altered macrophage M1 polarization while diminishing IL-17A-repressed M2 polarization, in part through a mechanism by which Wnt/β-catenin regulated JAK/STATs signaling-mediated macrophage polarization.

The role of Wnt/β-catenin signaling in development and diseases, as well as tissue homeostasis, has been well established (16). In addition to its role in T cell development and maturation (19), Wnt/β-catenin signaling also exhibited regulatory roles in immune responses in the pathogenesis of many diseases, such as pulmonary diseases, autoimmune diseases, and infectious diseases (20–22). Indeed, there is clinical and experimental evidence of activated Wnt/β-catenin signaling in lungs of patients with idiopathic pulmonary fibrosis (IPF) (23), and bleomycin-induced murine pulmonary fibrosis (24). In this context, Wnt/β-catenin is critical for recruitment of monocytes that are able to differentiate into macrophages in the injury site to modulate cell motility and adhesion (25). However, an excessive activation of Wnt/β-catenin signaling may induce IL-1β in alveolar epithelial cells and result in epithelial injury (26). In this regard, the alteration of M1 and M2 macrophage polarization may result in an imbalance of pro-inflammatory and anti-inflammatory mediators, both of which are involved in the pathogenesis of pulmonary fibrosis (27). There was evidence that Wnt/β-catenin signaling is involved in M1/M2 macrophage polarization in responses to varied stimuli (18). In this study, we also found that the Wnt3a-activated Wnt/β-catenin signaling inhibited M1 macrophage polarization in RAW264.7 cells. Interestingly the inhibition of Wnt/β-catenin signaling using small molecule XAV939 promoted M1 polarization but inhibited M2 macrophage phenotype. This finding was in disagreement with a result in a co-culture model of hepatic tumor cells and macrophages, in which activation of Wnt/β-catenin signaling in tumor cells induced an M2-like polarization of tumor-associated macrophages (28). Together with our findings, these results imply that Wnt/β-catenin signaling-altered macrophage polarization may occur within the cell context and be microenvironment-dependent; the precise mechanism underpinning Wnt/β-catenin signaling-regulated macrophage polarization remains to be determined.

In addition to its ability to alter macrophage polarization, Wnt/β-catenin signaling was able to interact with other cellular signaling or mediators, such as IL-17A, to modulate immune response and cell differentiation. As one of the best-studied members of the IL-17 family, IL-17A plays a crucial role in the development and progression of both acute and chronic inflammatory-related diseases (29). Similar to Wnt/β-catenin signaling, several lines of evidence have shown that IL-17A could promote macrophage M1 polarization (12). In line with these findings, our study also revealed that IL-17A induced M1 macrophage polarization in RAW264.7 cells. Interestingly, the IL-17A-induced M1 macrophage polarization could be inhibited by a Wnt3a-mediated activation of Wnt/β-catenin signaling in the naive state of RAW264.7 cells. This finding was consistent with findings of the ability of Wnt/β-catenin to promote Th2 cell differentiation (30), and its inhibitor secreted frizzled-related protein 1 (sFRP1) promotes Th17 cell differentiation (31). *Vice versa*, IL-17A was also able to activate Wnt/ β-catenin signaling in RAW264.7 cells. This result was inconsistent with a study in osteoblasts, where IL-17A inhibited Wnt signaling activity, and IL-17A-mediated inhibition of Wnt signaling contributed to bone loss in chronic skin inflammation (32). These results further suggest cell-context dependent interaction between Wnt signaling and IL-17A.

Mechanistically, macrophage polarization is tightly controlled by the activation of various transcriptional factors and cellular signaling pathways. Among them, JAK/STAT is one of the best-known pathways that play key roles in macrophage polarization. Indeed, previous studies have demonstrated that an up-regulation of Stat6 led the activation of Wnt signaling and induced M2 macrophage polarization (33). *Vice versa*, an overexpression of β-catenin could increase STAT3 expression and activate STAT signaling in lymphoma kinase-positive anaplastic large cell lymphoma (34). In this study, Wnt3a alone resulted in the activation of STAT1 signaling, and the XAV939-mediated inhibition of Wnt signaling led to an enhanced STAT3 and STAT6 activation. In addition, Wnt3a decreased the IL-17A-induced STAT3 activity, and inhibition of Wnt/β-catenin signaling further increased the IL-17A-induced STAT3 and STAT6 activation. These data might imply that Wnt/β-catenin signaling inhibited IL-17A-promoted M1 macrophage polarization by inhibiting STAT3/6 signaling, while diminishing IL-17A-suppressed M2 macrophage polarization by activating STAT1 signaling in RAW264.7 cells.

Collectively, this study revealed a novel function of Wnt/β-catenin signaling in regulating IL-17A-altered macrophage polarization.
